# On Some Nonclassical Algebraic Properties of Interval-Valued Fuzzy Soft Sets

**DOI:** 10.1155/2014/192957

**Published:** 2014-07-20

**Authors:** Xiaoyan Liu, Feng Feng, Hui Zhang

**Affiliations:** ^1^Department of Applied Mathematics, School of Science, Xi'an University of Posts and Telecommunications, Xi'an 710121, China; ^2^Mathematics and OR Section, Xi'an Research Institute of High-Tech Hongqing Town, Xi'an 710025, China

## Abstract

Interval-valued fuzzy soft sets realize a hybrid soft computing model in a general framework. Both Molodtsov's soft sets and interval-valued fuzzy sets can be seen as special cases of interval-valued fuzzy soft sets. In this study, we first compare four different types of interval-valued fuzzy soft subsets and reveal the relations among them. Then we concentrate on investigating some nonclassical algebraic properties of interval-valued fuzzy soft sets under the soft product operations. We show that some fundamental algebraic properties including the commutative and associative laws do not hold in the conventional sense, but hold in weaker forms characterized in terms of the relation =_*L*_. We obtain a number of algebraic inequalities of interval-valued fuzzy soft sets characterized by interval-valued fuzzy soft inclusions. We also establish the weak idempotent law and the weak absorptive law of interval-valued fuzzy soft sets using interval-valued fuzzy soft *J*-equal relations. It is revealed that the soft product operations ∧ and ∨ of interval-valued fuzzy soft sets do not always have similar algebraic properties. Moreover, we find that only distributive inequalities described by the interval-valued fuzzy soft *L*-inclusions hold for interval-valued fuzzy soft sets.

## 1. Introduction

In recent years, uncertainty modelling has become an important research topic in computational intelligence and related fields. It is worth noting that uncertainty is multifaceted and as such cannot be captured within a single mathematical framework. In response to this fact, a number of different approaches such as probability theory, fuzzy sets [1], and rough sets [2] have been developed, which are capable of addressing various types of uncertainties from differing viewpoints including randomness, gradualness, and granulation, respectively. Molodtsov [3] proposed* soft set theory* as a generic mathematical model for dealing with uncertainty from a parameterization point of view in about a decade ago. Since then there has been a rapid development in this theory and its applications to algebraic structures [4–10], topology [11, 12], knowledge acquisition [13], decision making [14], and others [15, 16]. Soft sets are also closely related to many other soft computing models [17–19]. Algebraic operations of soft sets were initiated by Molodtsov [3] and systematically investigated by Maji et al. [20]. Ali et al. [21] defined some new operations in soft set theory and further studied algebraic structures of soft sets associated with these new operations in [22].

On the other hand, some researchers considered extending soft sets with fuzzy set theory [23–27]. Yang et al. [25] introduce interval-valued fuzzy soft sets which realize a common extension of both Molodtsov's soft sets and interval-valued fuzzy sets. There is no doubt that soft subsets play a fundamental role in soft set theory. Maji et al. [20] initiated the notion of soft subsets in a very strict manner. Other types of soft subsets were also proposed by relaxing the conditions for defining Maji's soft subsets in [18, 28, 29]. Feng and Li [30] investigated these different types of soft subsets and the related soft equal relations in a systematic way. They also considered the free soft algebras associated with soft product operations.

The present study aims to investigate the above line of exploration in the more general setting of interval-valued fuzzy soft sets. We will investigate some nonclassical algebraic properties of interval-valued fuzzy soft sets with respect to soft product operations, which are distinct from those of interval-valued fuzzy sets. Note that some of the results obtained here are natural extensions to those corresponding results concerning Molodtsov's soft sets or Maji's fuzzy soft sets.

The remainder of the present paper is organized as follows.  Section 2 first recalls some basic notions related to interval-valued fuzzy soft sets.  Section 3 mainly discuss four types of interval-valued fuzzy soft subsets and the corresponding interval-valued fuzzy soft equal relations. Generalized commutative laws and generalized associative laws of interval-valued fuzzy soft sets are obtained as well. In Section 4, we propose many algebraic inequalities of IVF soft sets and use them to explore some nonclassical algebraic properties of interval-valued fuzzy soft sets with respect to soft product operations. Finally, the last section gives conclusions to complete this study and suggests potential directions for future work.

## 2. Preliminaries

Let *U* be an initial universe of objects and let *E*_*U*_ (or simply *E*) be the set of all parameters associated with objects in *U*, called a* parameter space*. In most cases parameters are considered to be attributes, characteristics, or properties of objects in *U*. The pair (*U*, *E*) is also known as a* soft universe*. We denote the power sets of *U* by *P*(*U*). The concept of soft sets is defined as follows.

**Definition 1 (see [3]).**

A pair *S* = (*F*, *A*) is called a* soft set* over *U*, where *A*⊆*E* and *F* : *A* → *P*(*U*) is a set-valued mapping, called the* approximate function* of the soft set *S*.

By means of parametrization, a soft set produces a series of approximate descriptions of a complicated object being perceived from various points of view. In other words, a soft set *S* = (*F*, *A*) over the universe *U* can be regarded as a parameterized family of subsets of the universe *U*. For any parameter *ϵ* ∈ *A*, the subset *F*(*ϵ*)⊆*U* may be interpreted as the set of *ϵ-approximate elements* [3]. Note that *F*(*ϵ*) may be arbitrary: some of them may be empty, and some may have nonempty intersections [3].

Maji et al. [23] initiated the study on hybrid structures involving both fuzzy sets and soft sets. They introduced the notion called fuzzy soft sets, which can be seen as a fuzzy generalization of Molodtsov's soft sets.

**Definition 2 (see [23]).**

Let *F*(*U*) be the set of all fuzzy subsets in a universe *U*. Let *E* be a set of parameters and *A*⊆*E*. A pair $\left(\tilde{F},A\right)$ is called a* fuzzy soft set* over *U*, where $\tilde{F}$ is a mapping given by $\tilde{F}:A\to F(U)$.

In the above definition, fuzzy subsets in the universe *U* are used as substitutes for the crisp subsets of *U*. Hence, it is clear that every (classical) soft set may be considered as a fuzzy soft set. Using the standard notations, the fuzzy set $\tilde{F}(\epsilon )$ can be written as $\tilde{F}(\epsilon )=\{(x,\tilde{F}(\epsilon )(x)):x\in U\}$.

Now we consider the set *L* ^*I*^ = {[*a*, *b*] : 0 ≤ *a* ≤ *b* ≤ 1} and the order relation ≤_*L*^*I*^_ given by

(1) [a1,b1]≤LI[a2,b2]⟺a1≤a2,  b1≤b2,          ∀[a1,b1],[a2,b2]∈LI.

Then *L* ^*I*^ = (*L* ^*I*^, ≤_*L*^*I*^_) is a complete lattice. An* interval-valued fuzzy set* on a universe *U* is a mapping *μ* : *U* → *L* ^*I*^. The union, intersection, and complement of interval-valued fuzzy sets can be obtained by canonically extending fuzzy set-theoretic operations to intervals. The set of all interval-valued fuzzy sets on *U* is denoted by *I*(*U*).

**Definition 3 (see [28]).**

Let (*U*, *E*) be a soft universe and *A*⊆*E*. A pair $I=(\tilde{F},A)$ is called an* interval-valued fuzzy soft set* over *U*, where $\tilde{F}$ is a mapping given by $\tilde{F}:A\to I(U)$.

It is easy to see that fuzzy soft sets are a special case of interval-valued fuzzy soft sets since interval-valued fuzzy sets are extensions of fuzzy sets. This ensures that the results obtained in this paper can easily be applied to fuzzy soft sets or Molodtsov's soft sets.

**Definition 4 (see [25]).**

Let $A=(\tilde{F},A)$ and $B=(\tilde{G},B)$ be two interval-valued fuzzy soft sets over *U*. The ∧*-product* (also called AND operation) of interval-valued fuzzy soft sets *A* and *B* is an interval-valued fuzzy soft set defined by *A*∧*B* = (*H*, *A* × *B*), where *H*(*x*, *y*) = *F*(*x*)∩*G*(*y*) for all (*x*, *y*) ∈ *A* × *B*.

**Definition 5 (see [25]).**

Let $A=(\tilde{F},A)$ and $B=(\tilde{G},B)$ be two interval-valued fuzzy soft sets over *U*. The ∨*-product* (also called OR operation) of interval-valued fuzzy soft sets *A* and *B* is an interval-valued fuzzy soft set defined by *A*∨*B* = (*H*, *A* × *B*), where *H*(*x*, *y*) = *F*(*x*) ∪ *G*(*y*) for all (*x*, *y*) ∈ *A* × *B*.

The above two operations ∧ and ∨ will be referred to as* soft product operations* of interval-valued fuzzy soft sets in general. We denote by *S* ^*I*^(*U*, *E*) the collection of all interval-valued fuzzy soft sets over *U* with parameter space *E*. For more details on interval-valued fuzzy soft sets and related terminologies used below, we refer to the papers [25, 28, 31].

## 3. Four Types of Interval-Valued Fuzzy Soft Subsets

For the sake of simplicity, the term “interval-valued fuzzy” will be abbreviated to IVF in what follows. The following type of soft subsets was first considered by Maji et al. [20] for Molodtsov's soft sets. Here we extend it to the more general seating of IVF soft sets.

**Definition 6 .**

Let $\left(\tilde{F},A\right)$ and $\left(\tilde{G},B\right)$ be two IVF soft sets over *U*. Then $\left(\tilde{F},A\right)$ is called a* IVF soft M-subset* of $\left(\tilde{G},B\right)$, denoted by $\left(\tilde{F},A)\;{\tilde{\subseteq }}_{M}\;(\tilde{G},B\right)$, if

1. *A*⊆*B*;
2. $\tilde{F}(a)=\tilde{G}(a)$ (i.e., $\tilde{F}(a)$ and $\tilde{G}(a)$ are identical approximations) for all *a* ∈ *A*.

Two IVF soft sets $\left(\tilde{F},A\right)$ and $\left(\tilde{G},B\right)$ are said to be* IVF soft M-equal*, denoted by $\left(\tilde{F},A)\;{=}_{M}\;(\tilde{G},B\right)$, if $\left(\tilde{F},A)\;{\tilde{\subseteq }}_{M}\;(\tilde{G},B\right)$ and $\left(\tilde{G},B)\;{\tilde{\subseteq }}_{M}\;(\tilde{F},A\right)$.

Another kind of IVF soft subsets was initiated by Yang et al. [25] in the following way.

**Definition 7 (see [25]).**

Let $\left(\tilde{F},A\right)$ and $\left(\tilde{G},B\right)$ be two IVF soft sets over *U*. Then $\left(\tilde{F},A\right)$ is called a* IVF soft F-subset* of $\left(\tilde{G},B\right)$, denoted by $\left(\tilde{F},A)\;{\tilde{\subseteq }}_{F}\;(\tilde{G},B\right)$, if *A*⊆*B* and $\tilde{F}(a)\subseteq \tilde{G}(a)$, for all *a* ∈ *A*.

Two IVF soft sets $\left(\tilde{F},A\right)$ and $\left(\tilde{G},B\right)$ are said to be* IVF soft F-equal*, denoted by $\left(\tilde{F},A)\;{=}_{F}\;(\tilde{G},B\right)$, if $\left(\tilde{F},A)\;{\tilde{\subseteq }}_{F}\;(\tilde{G},B\right)$ and $\left(\tilde{G},B)\;{\tilde{\subseteq }}_{F}\;(\tilde{F},A\right)$.

For two IVF soft sets $A=(\tilde{F},A)$ and $B=(\tilde{G},B)$, it is easy to see that

(2) $$\begin{array}{c} \left(\tilde{F},A\right){\tilde{\subseteq }}_{M}\left(\tilde{G},B\right)⟹\left(\tilde{F},A\right){\tilde{\subseteq }}_{F}\left(\tilde{G},B\right). \end{array}$$

However, the reverse implication may not be true as illustrated by the following example.

**Example 8 .**

Let *U* = {*u*_1_, *u*_2_, *u*_3_, *u*_4_, *u*_5_, *u*_6_} be the universe and the parameter space *E* = {*e*_1_, *e*_2_, *e*_3_, *e*_4_, *e*_5_}. For the parameter sets *A* = {*e*_1_, *e*_2_} and *B*_1_ = {*e*_1_, *e*_2_, *e*_3_}, let $A=(\tilde{F},A)$ and $B_{1}=(\tilde{G_{1}},B_{1})$ be two IVF soft sets over *U* whose tabular representations are given by Tables 1 and 2, respectively. Then it is easy to verify that $\left(\tilde{F},A)\;{\tilde{\subseteq }}_{F}\;(\tilde{G_{1}},B_{1}\right)$, but $\left(\tilde{F},A)\;{\tilde{\subseteq }}_{M}\;(\tilde{G_{1}},B_{1}\right)$ does not hold.

**Proposition 9 .**

Let $A=(\tilde{F},A)$ and $B=(\tilde{G},B)$ be two IVF soft sets over *U*. Then the following conditions are equivalent:

1. *A* =_*M*_ *B*;
2. *A* =_*F*_ *B*;
3. *A* = *B* and $\tilde{F}=\tilde{G}$.

**Proof**

It is straightforward and thus omitted.

By Proposition 9, we know that the IVF soft equal relations =_*M*_ and =_*F*_ coincide with each other. Hence, in what follows we write ≡ (called* IVF soft identical relation*) instead of =_*M*_ or =_*F*_ unless stated otherwise.

It is clear that ${\tilde{\subseteq }}_{M}$ and ${\tilde{\subseteq }}_{F}$ can be viewed as binary relations on the collection *S* ^*I*^(*U*, *E*) of all interval-valued fuzzy soft sets over *U* with parameter space *E*. From this point of view, ${\tilde{\subseteq }}_{M}$ and ${\tilde{\subseteq }}_{F}$ are also referred to as the* IVF soft M-inclusion* and* IVF soft F-inclusion* on *S* ^*I*^(*U*, *E*), respectively.

**Proposition 10 .**

The IVF soft F-inclusion ${\tilde{\subseteq }}_{F}$ is a partial order on *S* ^*I*^(*U*, *E*).

**Proof**

Let $\left(\tilde{F},A\right)$, $\left(\tilde{G},B\right)$, and $\left(\tilde{H},C\right)$ be IVF soft sets in *S* ^*I*^(*U*, *E*). First, it is easy to see that $\left(\tilde{F},A)\;{\tilde{\subseteq }}_{F}\;(\tilde{F},A\right)$; hence, ${\tilde{\subseteq }}_{F}$ is reflexive. Then assume that $\left(\tilde{F},A)\;{\tilde{\subseteq }}_{F}\;(\tilde{G},B\right)$ and $\left(\tilde{G},B)\;{\tilde{\subseteq }}_{F}\;(\tilde{F},A\right)$. By Proposition 9, it follows that *A* = *B* and $\tilde{F}=\tilde{G}$. That is, $\left(\tilde{F},A)\equiv (\tilde{G},B\right)$, which means the two IVF soft sets are identical. This shows that ${\tilde{\subseteq }}_{F}$ is antisymmetric. Finally, suppose that $\left(\tilde{F},A)\;{\tilde{\subseteq }}_{F}\;(\tilde{G},B\right)$ and $\left(\tilde{G},B)\;{\tilde{\subseteq }}_{F}\;(\tilde{H},C\right)$. Then by definition of IVF soft *F*-subsets, we have *A*⊆*B*⊆*C* and $\tilde{F}(a)\subseteq \tilde{G}(a)\subseteq \tilde{H}(a)$ for all *a* ∈ *A*. It follows that $\left(\tilde{F},A)\;{\tilde{\subseteq }}_{F}\;(\tilde{H},C\right)$ and so ${\tilde{\subseteq }}_{F}$ is transitive. Therefore, we conclude that the IVF soft *F*-inclusion ${\tilde{\subseteq }}_{F}$ is a partial order on *S* ^*I*^(*U*, *E*).

In a similar fashion, we can also verify that the IVF soft *M*-inclusion ${\tilde{\subseteq }}_{M}$ is a partial order on *S* ^*I*^(*U*, *E*).

**Proposition 11 .**

The IVF soft *M*-inclusion ${\tilde{\subseteq }}_{M}$ is a partial order on *S* ^*I*^(*U*, *E*).

**Proof**

The proof is similar to that of Proposition 10 and thus omitted.

Yang and Jun [28] proposed the following type of IVF soft subsets, which extends IVF soft *F*-subsets in a substantial way.

**Definition 12 (see [28]).**

Let $\left(\tilde{F},A\right)$ and $\left(\tilde{G},B\right)$ be two IVF soft sets over *U*. Then $\left(\tilde{F},A\right)$ is called a* IVF soft J-subset* of $\left(\tilde{G},B\right)$, denoted by $\left(\tilde{F},A)\;{\tilde{\subseteq }}_{J}\;(\tilde{G},B\right)$, if for every *a* ∈ *A* there exists *b* ∈ *B* such that $\tilde{F}(a)\subseteq \tilde{G}(b)$.

Two IVF soft sets $\left(\tilde{F},A\right)$ and $\left(\tilde{G},B\right)$ are said to be* IVF soft J-equal*, denoted by $\left(\tilde{F},A)\;{=}_{J}\;(\tilde{G},B\right)$, if $\left(\tilde{F},A)\;{\tilde{\subseteq }}_{J}\;(\tilde{G},B\right)$ and $\left(\tilde{G},B)\;{\tilde{\subseteq }}_{J}\;(\tilde{F},A\right)$.

Given two IVF soft sets $A=(\tilde{F},A)$ and $B=(\tilde{G},B)$ over *U*, one easily observes that $A\;{\tilde{\subseteq }}_{F}\;B$ implies $A\;{\tilde{\subseteq }}_{J}\;B$. However, the reverse may not be true as illustrated by the following example.

**Example 13 .**

Consider the two IVF soft sets $A=(\tilde{F},A)$ and $B_{2}=(\tilde{G_{2}},B_{2})$ shown in Tables 1 and 3. Then we can see that $A\;{\tilde{\subseteq }}_{J}\;B_{2}$ since $\tilde{F}(e_{1})\subseteq \tilde{G_{2}}(e_{3})$ and $\tilde{F}(e_{2})=\tilde{G_{2}}(e_{2})$, but $A\;{\tilde{\subseteq }}_{F}\;B_{2}$ does not hold since *A* ⊈ *B*_2_. Moreover, it is worth noting that the IVF soft sets *A* and *B*_2_ are not IVF soft *J*-equal since we see that *B*_2_ is not an IVF soft *J*-subset of *A*.

Motivated by Jun and Yang's IVF soft *J*-subsets, we further introduced a new kind of IVF soft subsets as follows.

**Definition 14 (see [29]).**

Let $\left(\tilde{F},A\right)$ and $\left(\tilde{G},B\right)$ be two IVF soft sets over *U*. Then $\left(\tilde{F},A\right)$ is called a* IVF soft L-subset* of $\left(\tilde{G},B\right)$, denoted by $\left(\tilde{F},A)\;{\tilde{\subseteq }}_{L}\;(\tilde{G},B\right)$, if for every *a* ∈ *A* there exists *b* ∈ *B* such that $\tilde{F}(a)=\tilde{G}(b)$.

Two IVF soft sets $\left(\tilde{F},A\right)$ and $\left(\tilde{G},B\right)$ are said to be* IVF soft L-equal*, denoted by $\left(\tilde{F},A)\;{=}_{L}\;(\tilde{G},B\right)$, if $\left(\tilde{F},A)\;{\tilde{\subseteq }}_{L}\;(\tilde{G},B\right)$ and $\left(\tilde{G},B)\;{\tilde{\subseteq }}_{L}\;(\tilde{F},A\right)$.

Obviously, ${\tilde{\subseteq }}_{J}$ and ${\tilde{\subseteq }}_{L}$ are binary relations on *S* ^*I*^(*U*, *E*), which are called the* IVF soft J-inclusion* and* IVF soft L-inclusion*, respectively.

**Proposition 15 .**

The IVF soft J-inclusion ${\tilde{\subseteq }}_{J}$ is a quasiorder on *S* ^*I*^(*U*, *E*).

**Proof**

Let $\left(\tilde{F},A\right)$, $\left(\tilde{G},B\right)$, and $\left(\tilde{H},C\right)$ be IVF soft sets in *S* ^*I*^(*U*, *E*). Note first that $\left(\tilde{F},A)\;{\tilde{\subseteq }}_{J}\;(\tilde{F},A\right)$ and so ${\tilde{\subseteq }}_{J}$ is reflexive. Next, suppose that $\left(\tilde{F},A)\;{\tilde{\subseteq }}_{J}\;(\tilde{G},B\right)$ and $\left(\tilde{G},B)\;{\tilde{\subseteq }}_{J}\;(\tilde{H},C\right)$. For every *a* ∈ *A*, there exists *b* ∈ *B* such that $\tilde{F}(a)\;\subseteq \;\tilde{G}(b)$ since $\left(\tilde{F},A\right)$ is an IVF soft *J*-subset of $\left(\tilde{G},B\right)$. Moreover, there exists *c* ∈ *C* such that $\tilde{G}(b)\;\subseteq \;\tilde{H}(c)$ since $\left(\tilde{G},B\right)$ is also an IVF soft *J*-subset of $\left(\tilde{H},C\right)$. It follows that $\tilde{F}(a)\;\subseteq \;\tilde{H}(c)$. Thus, we deduce that $\left(\tilde{F},A){\tilde{\subseteq }}_{J}(\tilde{H},C\right)$ and so ${\tilde{\subseteq }}_{J}$ is transitive. Therefore, the IVF soft *J*-inclusion ${\tilde{\subseteq }}_{J}$ is a quasiorder on *S* ^*I*^(*U*, *E*).

**Proposition 16 .**

The IVF soft L-inclusion ${\tilde{\subseteq }}_{L}$ is a quasiorder on *S* ^*I*^(*U*, *E*).

**Proof**

The proof is similar to that of Proposition 15 and thus omitted.

Suppose that $A=(\tilde{F},A)$ and $B=(\tilde{G},B)$ are two IVF soft sets over *U*. By definition, it can be verified that

(3) $$\begin{array}{c} A\;{\tilde{\subseteq }}_{M}\;B⟹A\;{\tilde{\subseteq }}_{L}\;B⟹A\;{\tilde{\subseteq }}_{J}\;B. \end{array}$$

However, it is worth noting that the reverse implications do not hold in general. Note also that the IVF soft *L*-inclusion ${\tilde{\subseteq }}_{L}$ and the IVF soft *J*-inclusion ${\tilde{\subseteq }}_{J}$ are only quasiorders instead of partial orders on *S* ^*I*^(*U*, *E*). In general, these relations are not antisymmetric. As illustration, we consider an example as follows.

**Example 17 .**

Consider the two IVF soft sets $A=(\tilde{F},A)$ and $B_{3}=(\tilde{G_{3}},B_{3})$ shown in Tables 1 and 4. Then we have $A\;{\tilde{\subseteq }}_{L}\;B_{3}$ since $\tilde{F}(e_{1})=\tilde{G_{3}}(e_{3})$ and $\tilde{F}(e_{2})=\tilde{G_{3}}(e_{2})$, but $A\;{\tilde{⊈}}_{M}\;B_{3}$ since *A* ⊈ *B*_3_. As shown in Example 13, we have $A\;{\tilde{\subseteq }}_{J}\;B_{2}$, but $A\;{\tilde{\subseteq }}_{L}\;B_{2}$ does not hold since $\tilde{F}(e_{1})\neq \tilde{G_{2}}(e_{2})$, $\tilde{F}(e_{1})\neq \tilde{G_{2}}(e_{3})$, and $\tilde{F}(e_{1})\neq \tilde{G_{2}}(e_{4})$. In addition, we can deduce that *A* and *B*_3_ are IVF soft *L*-equal since we also have $B_{3}\;{\tilde{\subseteq }}_{L}\;A$. However, it is clear that *A* ≡ *B*_3_ does not hold. This shows that the IVF soft *L*-inclusion ${\tilde{\subseteq }}_{L}$ is not antisymmetric. Similarly, one can verify that *A* =_*J*_ *B*_3_, which implies that the IVF soft *J*-inclusion ${\tilde{\subseteq }}_{J}$ is not antisymmetric. Thus, ${\tilde{\subseteq }}_{L}$ and ${\tilde{\subseteq }}_{J}$ are only quasiorders instead of partial orders.

**Remark 18 .**

**Figure 1:**
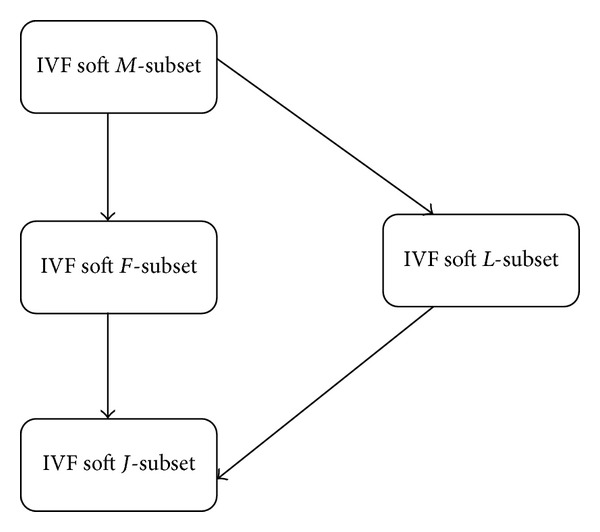
Relations among different types of IVF soft subsets.

In view of the above discussion, we have investigated four different types of soft subsets, including IVF soft *M*-subsets, IVF soft *F*-subsets, IVF soft *J*-subsets, and IVF soft L-subsets. In conclusion, Figure 1 illustrates the relations among different types of IVF soft subsets, in which a single arrow *A* → *B* denotes that *B* is a generalization of *A*. These results extend the corresponding results for Molodtsov's soft sets in [30].

As an immediate consequence of the above results, we also obtain the following relations among three types of IVF soft equal relations.

**Corollary 19 .**

Suppose that $A=(\tilde{F},A)$ and $B=(\tilde{G},B)$ are two IVF soft sets over *U*. Then we have

(4) $$\begin{array}{c} A\equiv B⟹A\;{=}_{L}\;B⟹A\;{=}_{J}\;B. \end{array}$$

It is worth noting that the IVF soft equal relations ≡, =_*L*_, and =_*J*_ are essentially distinct notions. For more details, one can refer to the discussion regarding these relations for Molodtsov's soft sets in [29].

At the end of this section, we characterize some basic algebraic properties of soft product operations ∧ and ∨ in virtue of IVF soft *L*-equal relations. First, we consider properties regarding commutativity of soft product operations.

**Theorem 20 .**

Let $\left(\tilde{F},A\right)$ and $\left(\tilde{G},B\right)$ be two IVF soft sets over *U*. Then we have

1. $\left(\tilde{F},A)\wedge (\tilde{G},B)\;{=}_{L}\;(\tilde{G},B)\wedge (\tilde{F},A\right)$;
2. $\left(\tilde{F},A)\vee (\tilde{G},B)\;{=}_{L}\;(\tilde{G},B)\vee (\tilde{F},A\right)$.

**Proof**

To prove the first assertion, let us write $\left(\tilde{L},A\times B\right)$ for $\left(\tilde{F},A)\wedge (\tilde{G},B\right)$, where $\tilde{L}(a,b)=\tilde{F}(a)\cap \tilde{G}(b)$ for all (*a*, *b*) ∈ *A* × *B*. We denote $\left(\tilde{G},B)\wedge (\tilde{F},A\right)$ by $\left(\tilde{R},B\times A\right)$, where $\tilde{R}(b,a)=\tilde{G}(b)\cap \tilde{F}(a)$ for all (*b*, *a*) ∈ *B* × *A*.

For any (*a*, *b*) ∈ *A* × *B*, there exists (*b*, *a*) ∈ *B* × *A* such that

(5) $$\begin{array}{c} \tilde{L}\left(a,b\right)=\tilde{F}\left(a\right)\cap \tilde{G}\left(b\right)=\tilde{G}\left(b\right)\cap \tilde{F}\left(a\right)=\tilde{R}\left(b,a\right). \end{array}$$

Hence, by definition of IVF soft *L*-subsets, we have $\left(\tilde{L},A\times B)\;{\tilde{\subseteq }}_{L}\;(\tilde{R},B\times A\right)$. The reverse IVF soft *L*-inclusion follows analogously. Therefore, we have

(6) $$\begin{array}{c} \left(\tilde{F},A\right)\wedge \left(\tilde{G},B\right){=}_{L}\left(\tilde{G},B\right)\wedge \left(\tilde{F},A\right). \end{array}$$

The second assertion can be proved in a similar fashion and thus omitted.

**Remark 21 .**

The above results are referred to as the* generalized commutative laws* of IVF soft sets due to the following reason. On one hand, it is obvious that the two IVF soft sets obtained by calculation from the left and right sides are distinct since they have different parameter sets. Thus, commutative laws do not hold in the conventional sense, which are characterized in virtue of the IVF soft identical relation ≡. On the other hand, we have shown that commutative laws are valid in a weaker sense, characterized by using IVF soft *L*-equal relations =_*L*_. Note that Theorem 20 extends Theorem 5.13 (called the* generalized soft commutative laws*) in [30].

Next, we investigate properties of soft product operations by considering associative laws.

**Theorem 22 .**

Let $\left(\tilde{F},A\right)$, $\left(\tilde{G},B\right)$, and $\left(\tilde{H},C\right)$ be IVF soft sets over *U*. Then we have

1. $\left(\tilde{F},A)\wedge ((\tilde{G},B)\wedge (\tilde{H},C))\;{=}_{L}\;((\tilde{F},A)\wedge (\tilde{G},B))\wedge (\tilde{H},C\right)$;
2. $\left(\tilde{F},A)\vee ((\tilde{G},B)\vee (\tilde{H},C))\;{=}_{L}\;((\tilde{F},A)\vee (\tilde{G},B))\vee (\tilde{H},C\right)$.

**Proof**

To prove the first assertion, we denote $\left(\tilde{G},B)\wedge (\tilde{H},C\right)$ by $\left(\tilde{T},B\times C\right)$, where $\tilde{T}(b,c)=\tilde{G}(b)\cap \tilde{H}(c)$ for all (*b*, *c*) ∈ *B* × *C*. Let $\left(\tilde{F},A)\wedge (\tilde{T},B\times C)=(\tilde{L},A\times (B\times C)\right)$, where $\tilde{L}(a,(b,c))=\tilde{F}(a)\cap \tilde{T}(b,c)=\tilde{F}(a)\cap (\tilde{G}(b)\cap \tilde{H}(c))$ for all (*a*, (*b*, *c*)) ∈ *A* × (*B* × *C*). On the other hand, let us write $\left(\tilde{F},A)\wedge (\tilde{G},B\right)$ as $\left(\tilde{K},A\times B\right)$, where $\tilde{K}(a,b)=\tilde{F}(a)\cap \tilde{G}(b)$ for all (*a*, *b*) ∈ *A* × *B*. Let $\left(\tilde{K},A\times B)\wedge (\tilde{H},C)=(\tilde{R},(A\times B)\times C\right)$, where $\tilde{R}((a,b),c)=\tilde{K}(a,b)\cap \tilde{H}(c)=(\tilde{F}(a)\cap \tilde{G}(b))\cap \tilde{H}(c)$ for all ((*a*, *b*), *c*)∈(*A* × *B*) × *C*.

Now, for every (*a*, (*b*, *c*)) ∈ *A* × (*B* × *C*), there exists ((*a*, *b*), *c*)∈(*A* × *B*) × *C* such that

(7) L~(a,(b,c))=F~(a)∩(G~(b)∩H~(c))=(F~(a)∩G~(b))∩H~(c)=R~((a,b),c).

Hence, by definition of IVF soft *L*-subsets, we have

(8) $$\begin{array}{c} \left(\tilde{L},A\times \left(B\times C\right)\right){\tilde{\subseteq }}_{L}\left(\tilde{R},\left(A\times B\right)\times C\right). \end{array}$$

The reverse IVF soft *L*-inclusion follows in a similar fashion. Finally, we conclude that $\left(\tilde{L},A\times (B\times C))\;{=}_{L}\;(\tilde{R},(A\times B)\times C\right)$. The second assertion can be proved in a similar fashion and thus omitted.

**Remark 23 .**

It is worth emphasizing that the two IVF soft sets obtained by calculation from the left and right sides are distinct since they have different parameter sets, which means that associative laws are not valid with respect to the IVF soft identical relation ≡. Nevertheless, from the above results one can see that associative laws hold in a weaker sense, which are characterized in terms of the IVF soft *L*-equal relation =_*L*_. Hence, we refer to the above results as the* generalized associative laws* of IVF soft sets. Note that Theorem 22 amends Theorem 2 (called the* associative law of interval-valued fuzzy soft sets*) in [25].

## 4. Some Algebraic Inequalities of IVF Soft Sets

### 4.1. Basic Inequalities of IVF Soft Sets

In this subsection, we first investigate some basic inequalities of IVF soft sets characterized by IVF soft *J*-inclusions, which are useful in subsequent discussions.

**Proposition 24 .**

Let $\left(\tilde{F},A\right)$ and $\left(\tilde{G},B\right)$ be IVF soft sets over *U*. Then we have

1. $\left(\tilde{F},A){\tilde{\subseteq }}_{J}(\tilde{F},A)\vee (\tilde{G},B\right)$;
2. $\left(\tilde{G},B){\tilde{\subseteq }}_{J}(\tilde{F},A)\vee (\tilde{G},B\right)$.

**Proof**

To prove the first assertion, we write $\left(\tilde{F},A)\vee (\tilde{G},B\right)$ as $\left(\tilde{R},A\times B\right)$ with $\tilde{R}(a,b)=\tilde{F}(a)\cup \tilde{G}(b)$ for all (*a*, *b*) ∈ *A* × *B*. For every *a* ∈ *A*, let us choose any *b* ∈ *B* and then we get (*a*, *b*) ∈ *A* × *B* such that

(9) $$\begin{array}{c} \tilde{F}\left(a\right)\subseteq \tilde{R}\left(a,b\right)=\tilde{F}\left(a\right)\cup \tilde{G}\left(b\right). \end{array}$$

By definition of IVF soft *J*-subsets, we have $\left(\tilde{F},A){\tilde{\subseteq }}_{J}(\tilde{F},A)\vee (\tilde{G},B\right)$. The second assertion can be proved in an analogous fashion and thus omitted.

**Proposition 25 .**

Let $\left(\tilde{F},A\right)$ and $\left(\tilde{G},B\right)$ be IVF soft sets over *U*. Then we have

1. $\left(\tilde{F},A)\wedge (\tilde{G},B){\tilde{\subseteq }}_{J}(\tilde{F},A\right)$;
2. $\left(\tilde{F},A)\wedge (\tilde{G},B){\tilde{\subseteq }}_{J}(\tilde{G},B\right)$.

**Proof**

We only show the validity of (1); then the assertion (2) can be proved in a similar way. Let $\left(\tilde{F},A)\wedge (\tilde{G},B)=(\tilde{L},A\times B\right)$, where $\tilde{L}(a,b)=\tilde{F}(a)\cap \tilde{G}(b)$ for all (*a*, *b*) ∈ *A* × *B*. For every (*a*, *b*) ∈ *A* × *B*, it is easy to see that there exists *a* ∈ *A* such that $\tilde{L}(a,b)=\tilde{F}(a)\cap \tilde{G}(b)\subseteq \tilde{F}(a)$. By definition of IVF soft *J*-subsets, we have $\left(\tilde{L},A\times B)\;{\tilde{\subseteq }}_{J}\;(\tilde{F},A\right)$. That is, $\left(\tilde{F},A)\wedge (\tilde{G},B)\;{\tilde{\subseteq }}_{J}\;(\tilde{F},A\right)$.

As an immediate consequence of the above results, we get an inequality of IVF soft sets as follows.

**Corollary 26 .**

Let $\left(\tilde{F},A\right)$ and $\left(\tilde{G},B\right)$ be IVF soft sets over *U*. Then we have $\left(\tilde{F},A)\wedge (\tilde{G},B){\tilde{\subseteq }}_{J}(\tilde{G},B)\vee (\tilde{F},A\right)$.

Another similar but different inequality of IVF soft sets can be verified by using the definition of IVF soft *F*-subsets.

**Proposition 27 .**

Let $\left(\tilde{F},A\right)$ and $\left(\tilde{G},B\right)$ be IVF soft sets over *U*. Then we have $\left(\tilde{F},A)\wedge (\tilde{G},B)\;{\tilde{\subseteq }}_{F}\;(\tilde{F},A)\vee (\tilde{G},B\right)$.

**Proposition 28 .**

Let $\left(\tilde{F},A\right)$, $\left(\tilde{G},B\right)$, and $\left(\tilde{H},C\right)$ be IVF soft sets over *U*. If $\left(\tilde{F},A)\;{\tilde{\subseteq }}_{J}\;(\tilde{G},B\right)$, then we have

1. $\left(\tilde{F},A)\wedge (\tilde{H},C)\;{\tilde{\subseteq }}_{J}\;(\tilde{G},B)\wedge (\tilde{H},C\right)$;
2. $\left(\tilde{F},A)\wedge (\tilde{H},C)\;{\tilde{\subseteq }}_{J}\;(\tilde{H},C)\wedge (\tilde{G},B\right)$.

**Proof**

To prove the first assertion, let $\left(\tilde{F},A)\wedge (\tilde{H},C)=(\tilde{L},A\times C\right)$ and $\left(\tilde{G},B)\wedge (\tilde{H},C)=({\tilde{R}}_{1},B\times C\right)$. By hypothesis, we have $\left(\tilde{F},A)\;{\tilde{\subseteq }}_{J}\;(\tilde{G},B\right)$ and so for every *a* ∈ *A* there exists *b* ∈ *B* such that $\tilde{F}(a)\subseteq \tilde{G}(b)$. Now for any (*a*, *c*) ∈ *A* × *C*, we deduce that

(10) $$\begin{array}{c} \tilde{L}\left(a,c\right)=\tilde{F}\left(a\right)\cap \tilde{H}\left(c\right)\subseteq \tilde{G}\left(b\right)\cap \tilde{H}\left(c\right)={\tilde{R}}_{1}\left(b,c\right), \end{array}$$

for some (*b*, *c*) ∈ *B* × *C*. Hence, $\left(\tilde{L},A\times C)\;{\tilde{\subseteq }}_{J}\;({\tilde{R}}_{1},B\times C\right)$.

Next, we prove the second assertion. Let $\left(\tilde{H},C)\wedge (\tilde{G},B)=({\tilde{R}}_{2},C\times B\right)$. Note that we have already obtained that $\left(\tilde{L},A\times C)\;{\tilde{\subseteq }}_{J}\;({\tilde{R}}_{1},B\times C\right)$. By the generalized commutative laws of IVF soft sets shown in Theorem 20, we also deduce that

(11) (R~1,B×C)≡(G~,B)∧(H~,C)=L(H~,C)∧(G~,B)≡(R~2,C×B).

In particular, it follows that $\left({\tilde{R}}_{1},B\times C)\;{\tilde{\subseteq }}_{J}\;({\tilde{R}}_{2},C\times B\right)$. Since the IVF soft *J*-inclusion ${\tilde{\subseteq }}_{J}$ is a quasiorder on *S* ^*I*^(*U*, *E*), we deduce that

(12) $$\begin{array}{c} \left(\tilde{L},A\times C\right){\tilde{\subseteq }}_{J}\left({\tilde{R}}_{1},B\times C\right){\tilde{\subseteq }}_{J}\left({\tilde{R}}_{2},C\times B\right). \end{array}$$

That is, $\left(\tilde{F},A)\wedge (\tilde{H},C){\tilde{\subseteq }}_{J}(\tilde{H},C)\wedge (\tilde{G},B\right)$.

**Proposition 29 .**

Let $\left(\tilde{F},A\right)$, $\left(\tilde{G},B\right)$, and $\left(\tilde{H},C\right)$ be IVF soft sets over *U*. If $\left(\tilde{F},A){\tilde{\subseteq }}_{J}(\tilde{G},B\right)$, then we have

1. $\left(\tilde{H},C)\wedge (\tilde{F},A)\;{\tilde{\subseteq }}_{J}\;(\tilde{H},C)\wedge (\tilde{G},B\right)$;
2. $\left(\tilde{H},C)\wedge (\tilde{F},A)\;{\tilde{\subseteq }}_{J}\;(\tilde{G},B)\wedge (\tilde{H},C\right)$.

**Proof**

The proof is similar to that of Proposition 28 and thus omitted.

**Proposition 30 .**

Let $\left({\tilde{F}}_{i},A_{i}\right)$, $\left({\tilde{G}}_{i},B_{i}\right)$, be IVF soft sets over *U* and *i* = 1,2. If $\left({\tilde{F}}_{1},A_{1})\;{\tilde{\subseteq }}_{J}\;({\tilde{G}}_{1},B_{1}\right)$ and $\left({\tilde{F}}_{2},A_{2})\;{\tilde{\subseteq }}_{J}\;({\tilde{G}}_{2},B_{2}\right)$, then we have

(13) $$\begin{array}{c} \left({\tilde{F}}_{1},A_{1}\right)\wedge \left({\tilde{F}}_{2},A_{2}\right){\tilde{\subseteq }}_{J}\left({\tilde{G}}_{1},B_{1}\right)\wedge \left({\tilde{G}}_{2},B_{2}\right). \end{array}$$

**Proof**

Since $\left({\tilde{F}}_{1},A_{1})\;{\tilde{\subseteq }}_{J}\;({\tilde{G}}_{1},B_{1}\right)$, we deduce that

(14) $$\begin{array}{c} \left({\tilde{F}}_{1},A_{1}\right)\wedge \left({\tilde{G}}_{2},B_{2}\right){\tilde{\subseteq }}_{J}\left({\tilde{G}}_{1},B_{1}\right)\wedge \left({\tilde{G}}_{2},B_{2}\right), \end{array}$$

by the first assertion of Proposition 28. But we also have $\left({\tilde{F}}_{2},A_{2}){\tilde{\subseteq }}_{J}({\tilde{G}}_{2},B_{2}\right)$, which implies that

(15) $$\begin{array}{c} \left({\tilde{F}}_{1},A_{1}\right)\wedge \left({\tilde{F}}_{2},A_{2}\right){\tilde{\subseteq }}_{J}\left({\tilde{F}}_{1},A_{1}\right)\wedge \left({\tilde{G}}_{2},B_{2}\right), \end{array}$$

by the first assertion of Proposition 29. Since the IVF soft *J*-inclusion ${\tilde{\subseteq }}_{J}$ is a quasiorder on *S* ^*I*^(*U*, *E*), we finally conclude that

(16) $$\begin{array}{c} \left({\tilde{F}}_{1},A_{1}\right)\wedge \left({\tilde{F}}_{2},A_{2}\right){\tilde{\subseteq }}_{J}\left({\tilde{G}}_{1},B_{1}\right)\wedge \left({\tilde{G}}_{2},B_{2}\right), \end{array}$$

which completes the proof.

By considering ∨-product of IVF soft sets, we can get the following results which are analogous to Propositions 28, 29, and 30, respectively.

**Proposition 31 .**

Let $\left(\tilde{F},A\right)$, $\left(\tilde{G},B\right)$, and $\left(\tilde{H},C\right)$ be IVF soft sets over *U*. If $\left(\tilde{F},A)\;{\tilde{\subseteq }}_{J}\;(\tilde{G},B\right)$, then we have

1. $\left(\tilde{F},A)\vee (\tilde{H},C)\;{\tilde{\subseteq }}_{J}\;(\tilde{G},B)\vee (\tilde{H},C\right)$;
2. $\left(\tilde{F},A)\vee (\tilde{H},C)\;{\tilde{\subseteq }}_{J}\;(\tilde{H},C)\vee (\tilde{G},B\right)$.

**Proposition 32 .**

Let $\left(\tilde{F},A\right)$, $\left(\tilde{G},B\right)$, and $\left(\tilde{H},C\right)$ be IVF soft sets over *U*. If $\left(\tilde{F},A)\;{\tilde{\subseteq }}_{J}\;(\tilde{G},B\right)$, then we have

1. $\left(\tilde{H},C)\vee (\tilde{F},A)\;{\tilde{\subseteq }}_{J}\;(\tilde{H},C)\vee (\tilde{G},B\right)$;
2. $\left(\tilde{H},C)\vee (\tilde{F},A)\;{\tilde{\subseteq }}_{J}\;(\tilde{G},B)\vee (\tilde{H},C\right)$.

**Proposition 33 .**

Let $\left({\tilde{F}}_{i},A_{i}\right)$, $\left({\tilde{G}}_{i},B_{i}\right)$ be IVF soft sets over *U* and *i* = 1,2. If $\left({\tilde{F}}_{1},A_{1})\;{\tilde{\subseteq }}_{J}\;({\tilde{G}}_{1},B_{1}\right)$ and $\left({\tilde{F}}_{2},A_{2})\;{\tilde{\subseteq }}_{J}\;({\tilde{G}}_{2},B_{2}\right)$, then we have

(17) $$\begin{array}{c} \left({\tilde{F}}_{1},A_{1}\right)\vee \left({\tilde{F}}_{2},A_{2}\right){\tilde{\subseteq }}_{J}\left({\tilde{G}}_{1},B_{1}\right)\vee \left({\tilde{G}}_{2},B_{2}\right). \end{array}$$

### 4.2. Idempotent Inequality of IVF Soft Sets

In this subsection, we consider algebraic properties regarding idempotency of soft product operations and show that IVF soft sets have some nonclassical algebraic properties, compared with interval-valued fuzzy sets.

**Proposition 34 .**

Let $\left(\tilde{F},A)\in S^{I}(U,E\right)$. Then $\left(\tilde{F},A)\;{\tilde{\subseteq }}_{L}\;(\tilde{F},A)\vee (\tilde{F},A\right)$.

**Proof**

Assume that $\left(\tilde{F},A\right)$ is an IVF soft set over *U*. Let us denote $\left(\tilde{F},A)\vee (\tilde{F},A\right)$ by $\left(\tilde{R},A\times A\right)$, where $\tilde{R}(a_{1},a_{2})=\tilde{F}(a_{1})\cup \tilde{F}(a_{2})$ for all (*a*_1_, *a*_2_) ∈ *A* × *A*. For every *a* ∈ *A*, there exists (*a*, *a*) ∈ *A* × *A* such that

(18) $$\begin{array}{c} \tilde{F}\left(a\right)=\tilde{F}\left(a\right)\cup \tilde{F}\left(a\right)=\tilde{R}\left(a,a\right). \end{array}$$

By definition of IVF soft *L*-subsets, we have $\left(\tilde{F},A)\;{\tilde{\subseteq }}_{L}\;(\tilde{F},A)\vee (\tilde{F},A\right)$.

**Proposition 35 .**

Let $\left(\tilde{F},A)\in S^{I}(U,E\right)$. Then $\left(\tilde{F},A)\;{\tilde{\subseteq }}_{L}\;(\tilde{F},A)\wedge (\tilde{F},A\right)$.

**Proof**

The proof is similar to that of Proposition 34 and thus omitted.

**Proposition 36 .**

Let $\left(\tilde{F},A)\in S^{I}(U,E\right)$. Then $\left(\tilde{F},A)\wedge (\tilde{F},A\;){\tilde{\subseteq }}_{J}\;(\tilde{F},A\right)$.

**Proof**

This follows directly from Proposition 25 if the two IVF soft sets $\left(\tilde{F},A\right)$ and $\left(\tilde{G},B\right)$ are chosen to be identical.

**Theorem 37 .**

Let $\left(\tilde{F},A)\in S^{I}(U,E\right)$. Then $\left(\tilde{F},A)\;{=}_{J}\;(\tilde{F},A)\wedge (\tilde{F},A\right)$.

**Proof**

By Proposition 36, $\left(\tilde{F},A)\wedge (\tilde{F},A)\;{\tilde{\subseteq }}_{J}\;(\tilde{F},A\right)$. It suffices to show the reverse IVF soft *J*-inclusion. In fact, we have $\left(\tilde{F},A)\;{\tilde{\subseteq }}_{L}\;(\tilde{F},A)\wedge (\tilde{F},A\right)$ by Proposition 35. Using the conclusion in Remark 18, we immediately obtain $\left(\tilde{F},A)\;{\tilde{\subseteq }}_{J}\;(\tilde{F},A)\wedge (\tilde{F},A\right)$, and so $\left(\tilde{F},A)\;{=}_{J}\;(\tilde{F},A)\wedge (\tilde{F},A\right)$.

The above result (called the* weak idempotent law* of IVF soft sets) indicates that the ∧-product operation of IVF soft sets is idempotent with respect to IVF soft *J*-equal relations. On the other hand, it should be noted that we cannot form the parallel result for the ∨-product operation of IVF soft sets as illustrated by the following example.

**Example 38 .**

Let $\left(\tilde{F},A\right)$ be the IVF soft set given by Table 1 (see Example 8). Let $\left(\tilde{R},A\times A)=(\tilde{F},A)\vee (\tilde{F},A\right)$. By calculation, we get

(19) R~(e1,e2)=R~(e2,e1)={(u1,[0.4,0.7]),(u2,[0.2,0.6]),(u3,[0.5,0.7]), (u4,[0.8,0.9]),(u5,[0.6,0.7]), (u6,[0.4,0.6])},

and $\tilde{R}(e_{i},e_{i})=\tilde{F}(e_{i})$ (*i* = 1,2). It is easy to observe that $\tilde{R}(e_{1},e_{2})\;⊈\;\tilde{F}(e_{1})$ and $\tilde{R}(e_{1},e_{2})\;⊈\;\tilde{F}(e_{2})$. Hence, $\left(\tilde{R},A\times A)\;{\tilde{\subseteq }}_{J}\;(\tilde{F},A\right)$ does not hold. In particular, we further deduce that $\left(\tilde{F},A)\;\neq_{J}\;(\tilde{F},A)\vee (\tilde{F},A\right)$.

Moreover, let $\left({\tilde{R}}^{\prime },A\times A)=(\tilde{F},A)\wedge (\tilde{F},A\right)$. By calculation, we have

(20) R~′(e1,e2)=R~′(e2,e1)={(u1,[0.2,0.6]),(u2,[0.1,0.4]),(u3,[0.1,0.2]), (u4,[0.6,0.9]),(u5,[0.3,0.5]), (u6,[0.4,0.5])},

and ${\tilde{R}}^{\prime }(e_{i},e_{i})=\tilde{F}(e_{i})$ (*i* = 1,2). Then one can see that ${\tilde{R}}^{\prime }(e_{1},e_{2})\neq \tilde{F}(e_{1})$ and ${\tilde{R}}^{\prime }(e_{1},e_{2})\neq \tilde{F}(e_{2})$. This implies that $\left({\tilde{R}}^{\prime },A\times A)\;{\tilde{\subseteq }}_{L}\;(\tilde{F},A\right)$ do not hold and so we deduce that $\left(\tilde{F},A)\;\neq_{L}\;(\tilde{F},A)\wedge (\tilde{F},A\right)$.

**Remark 39 .**

The above results indicate that IVF soft sets have some nonclassical algebraic properties, compared with interval-valued fuzzy sets. By Definitions 4 and 5, the operations ∧ and ∨ of IVF soft sets are, respectively, defined by means of the intersection ∩ and union ∪ of interval-valued fuzzy sets. In the theory of interval-valued fuzzy sets, operations ∩ and ∪ are both idempotent since (*I*(*U*), ∩, ∪) is a lattice. Furthermore, we know that ∩ and ∪ are dual to each other, which always satisfy similar or parallel algebraic properties. Nevertheless, from Theorem 37 and Example 38, one can observe that the operations ∧ and ∨ of IVF soft sets do not always have similar algebraic properties. In fact, as shown above we have

(21) $$\begin{array}{c} \left(\tilde{F},A\right)\neq_{J}\left(\tilde{F},A\right)\vee \left(\tilde{F},A\right), \end{array}$$

while

(22) $$\begin{array}{c} \left(\tilde{F},A\right){=}_{J}\left(\tilde{F},A\right)\wedge \left(\tilde{F},A\right). \end{array}$$

It is also worth noting that

(23) $$\begin{array}{c} \left(\tilde{F},A\right)\neq_{L}\left(\tilde{F},A\right)\wedge \left(\tilde{F},A\right), \end{array}$$

which means that the ∧-product operation of IVF soft sets is idempotent with respect to the IVF soft *J*-equal relation =_*J*_, but not in the stronger sense of =_*L*_. Thus, by using the IVF soft *L*-inclusion ${\tilde{\subseteq }}_{L}$, we only have some idempotent inequalities shown in Propositions 34 and 35.

### 4.3. Distributive Inequality of IVF Soft Sets

In the above discussion, we have shown some nonclassical algebraic properties concerning soft product operations of IVF soft sets by considering idempotent laws. Next, we will investigate other interesting properties with regard to operations ∧ and ∨ of IVF soft sets by considering distributive laws.

**Theorem 40 .**

Let $\left(\tilde{F},A\right)$, $\left(\tilde{G},B\right)$, and $\left(\tilde{H},C\right)$ be IVF soft sets over *U*. Then we have

1. $\left((\tilde{F},A)\vee (\tilde{G},B))\wedge (\tilde{H},C)\;{\tilde{\subseteq }}_{L}\;((\tilde{F},A)\wedge (\tilde{H},C))\vee ((\tilde{G},B)\wedge (\tilde{H},C)\right)$;
2. $\left((\tilde{F},A)\wedge (\tilde{G},B))\vee (\tilde{H},C)\;{\tilde{\subseteq }}_{L}\;((\tilde{F},A)\vee (\tilde{H},C))\wedge ((\tilde{G},B)\vee (\tilde{H},C)\right)$.

**Proof**

We only show the validity of (1); then assertion (2) can be proved in a similar way. Let us write $\left(\tilde{F},A)\vee (\tilde{G},B\right)$ as $\left(\tilde{T},A\times B\right)$, where $\tilde{T}(a,b)=\tilde{F}(a)\cup \tilde{G}(b)$ for all (*a*, *b*) ∈ *A* × *B*. Then let $\left((\tilde{F},A)\vee (\tilde{G},B))\wedge (\tilde{H},C)=(\tilde{L},(A\times B)\times C\right)$, where

(24) $$\begin{array}{c} \tilde{L}\left(\left(a,b\right),c\right)=\tilde{T}\left(a,b\right)\cap \tilde{H}\left(c\right)=\left(\tilde{F}\left(a\right)\cup \tilde{G}\left(b\right)\right)\cap \tilde{H}\left(c\right) \end{array}$$

for all ((*a*, *b*), *c*)∈(*A* × *B*) × *C*.

Next, we write $\left(\tilde{M},A\times C\right)$ for $\left(\tilde{F},A)\wedge (\tilde{H},C\right)$, where $\tilde{M}(a,c)=\tilde{F}(a)\cap \tilde{H}(c)$ for all (*a*, *c*) ∈ *A* × *C*. Also let us write $\left(\tilde{N},B\times C\right)$ for $\left(\tilde{G},B)\wedge (\tilde{H},C\right)$, where $\tilde{N}(b,c)=\tilde{G}(b)\cap \tilde{H}(c)$ for all (*b*, *c*) ∈ *B* × *C*. Now let $\left(\tilde{M},A\times C)\vee (\tilde{N},B\times C)=(\tilde{R},(A\times C)\times (B\times C)\right)$, where

(25) R~((a,c),(b,c′))=M~(a,c)∪N~(b,c′)=(F~(a)∩H~(c))∪(G~(b)∩H~(c′))

for all ((*a*, *c*), (*b*, *c*′))∈(*A* × *C*)×(*B* × *C*).

For every ((*a*, *b*), *c*)∈(*A* × *B*) × *C*, there exists ((*a*, *c*), (*b*, *c*))∈(*A* × *C*)×(*B* × *C*) such that

(26) L~((a,b),c)=(F~(a)∪G~(b))∩H~(c)=(F~(a)∩H~(c))∪(G~(b)∩H~(c))=R~((a,c),(b,c)).

By Definition 14, $\left(\tilde{L},(A\times B)\times C)\;{\tilde{\subseteq }}_{L}\;(\tilde{R},(A\times C)\times (B\times C)\right)$. That is,

(27) ((F~,A)∨(G~,B))∧(H~,C)⊆~L((F~,A)∧(H~,C))∨((G~,B)∧(H~,C)),

completing the proof of (1).

In a similar fashion, one can verify the following distributive inequalities of IVF soft sets given by Liu et al. [29] without proofs.

**Theorem 41 .**

Let $\left(\tilde{F},A\right)$, $\left(\tilde{G},B\right)$, and $\left(\tilde{H},C\right)$ be IVF soft sets over *U*. Then we have

1. $\left(\tilde{F},A)\wedge ((\tilde{G},B)\vee (H,C)){\tilde{\subseteq }}_{L}((\tilde{F},A)\wedge (\tilde{G},B))\vee ((\tilde{F},A)\wedge (\tilde{H},C)\right)$;
2. $\left(\tilde{F},A)\vee ((\tilde{G},B)\wedge (\tilde{H},C)){\tilde{\subseteq }}_{L}((\tilde{F},A)\vee (\tilde{G},B))\wedge ((\tilde{F},A)\vee (\tilde{H},C)\right)$.

**Remark 42 .**

It is interesting to note that the operations ∧ and ∨ of IVF soft sets show some nonclassical algebraic properties when considering distributive laws. In the theory of interval-valued fuzzy sets, the inclusion relation ⊆ is a partial order on the set *I*(*U*) of all interval-valued fuzzy sets on a given universe *U* and we also know that (*I*(*U*), ∩, ∪) forms a distributive lattice. Thus, the operations ∩ and ∪ of interval-valued fuzzy sets satisfy both the left and the right distributive laws.

However, it can be found that only distributive inequalities (described by the IVF soft *L*-inclusion ${\tilde{\subseteq }}_{L}$, which is a quasiorder on *S* ^*I*^(*U*, *E*)) hold for IVF soft sets. In fact, neither the left nor the right distributive laws hold even in the weakest sense of IVF soft *J*-equal relations. To illustrate this, we consider an example as follows.

**Example 43 .**

For the parameter sets *A* = {*e*_1_, *e*_2_}, *B* = {*e*_3_}, and *C* = {*e*_4_}, let $A=(\tilde{F},A)$, $B=(\tilde{G},B)$, and $C=(\tilde{H},C)$ be three IVF soft sets over *U* as shown in Tables 1, 5, and 6. Let us write $\left(\tilde{T},B\times C\right)$ for $\left(\tilde{G},B)\vee (\tilde{H},C\right)$, where $\tilde{T}(b,c)=\tilde{G}(b)\cup \tilde{H}(c)$ for all (*b*, *c*) ∈ *B* × *C*. Then let $\left(\tilde{F},A)\wedge (\tilde{T},B\times C)=(\tilde{L},A\times (B\times C)\right)$, where

(28) $$\begin{array}{c} \tilde{L}\left(a,\left(b,c\right)\right)=\tilde{F}\left(a\right)\cap \tilde{T}\left(b,c\right)=\tilde{F}\left(a\right)\cap \left(\tilde{G}\left(b\right)\cup \tilde{H}\left(c\right)\right) \end{array}$$

for all (*a*, (*b*, *c*)) ∈ *A* × (*B* × *C*). It is easy to see that

(29) $$\begin{array}{c} A\times \left(B\times C\right)=\left\{\left(e_{1},\left(e_{3},e_{4}\right)\right),\left(e_{2},\left(e_{3},e_{4}\right)\right)\right\}. \end{array}$$

By calculation we obtain

(30) L~(e1,(e3,e4))=F~(e1)∩(G~(e3)∪H~(e4))={(u1,[0.3,0.5]),(u2,[0.2,0.6]), (u3,[0.1,0.2]),(u4,[0.7,0.9]), (u5,[0.3,0.5]),(u6,[0.4,0.6])},L~(e2,(e3,e4))=F~(e2)∩(G~(e3)∪H~(e4))={(u1,[0.2,0.5]),(u2,[0.1,0.4]), (u3,[0.3,0.5]),(u4,[0.6,0.9]), (u5,[0.4,0.6]),(u6,[0.4,0.5])}.

Next, we write $\left(\tilde{M},A\times B\right)$ for $\left(\tilde{F},A)\wedge (\tilde{G},B\right)$, where $\tilde{M}(a,b)=\tilde{F}(a)\cap \tilde{G}(b)$ for all (*a*, *b*) ∈ *A* × *B*. Also let us write $\left(\tilde{N},A\times C\right)$ for $\left(\tilde{F},A)\wedge (\tilde{H},C\right)$, where $\tilde{N}(a,c)=\tilde{F}(a)\cap \tilde{H}(c)$ for all (*a*, *c*) ∈ *A* × *C*. Now let $\left(\tilde{M},A\times B)\vee (\tilde{F},A\times C)=(\tilde{R},(A\times B)\times (A\times C)\right)$, where

(31) R~((a,b),(a′,c))=M~(a,b)∪N~(a′,c)=(F~(a)∩G~(b))∪(F~(a′)∩H~(c)),

for all ((*a*, *b*), (*a*′, *c*))∈(*A* × *B*)×(*A* × *C*). One easily see that *A* × *B* = {(*e*_1_, *e*_3_), (*e*_2_, *e*_3_)} and *A* × *C* = {(*e*_1_, *e*_4_), (*e*_2_, *e*_4_)}. Thus, we have

(32) (A×B)×(A×C) ={((e1,e3),(e1,e4)),((e1,e3),(e2,e4)),   ((e2,e3),(e1,e4)),((e2,e3),(e2,e4))}.

By calculation we obtain

(33) R~((e1,e3),(e2,e4)) =(F~(e1)∩G~(e3))∪(F~(e2)∩H~(e4)) ={(u1,[0.3,0.5]),(u2,[0.2,0.6]),(u3,[0.3,0.5]),   (u4,[0.6,0.9]),(u5,[0.3,0.6]),(u6,[0.4,0.5])},R~((e2,e3),(e1,e4)) =(F~(e2)∩G~(e3))∪(F~(e1)∩H~(e4)) ={(u1,[0.3,0.5]),(u2,[0.2,0.5]),(u3,[0.3,0.5]),   (u4,[0.7,0.9]),(u5,[0.4,0.5]),(u6,[0.4,0.6])}.

Clearly, we have $\tilde{R}((e_{1},e_{3}),(e_{2},e_{4}))\;⊈\;\tilde{L}(e_{1},(e_{3},e_{4}))$, $\tilde{R}((e_{1},e_{3}),(e_{2},e_{4}))\;⊈\;\tilde{L}(e_{2},(e_{3},e_{4}))$, $\tilde{R}((e_{2},e_{3}),(e_{1},e_{4}))\;⊈\;\tilde{L}(e_{1},(e_{3},e_{4}))$, and $\tilde{R}((e_{2},e_{3}),(e_{1},e_{4}))\;⊈\;\tilde{L}(e_{2},(e_{3},e_{4}))$. Therefore, $\left(\tilde{R},(A\times B)\times (A\times C))\;{\tilde{⊈}}_{J}\;(\tilde{L},A\times (B\times C)\right)$. This shows that

(34) (F~,A)∧((G~,B)∨(H~,C))=J((F~,A)∧(G~,B))∨((F~,A)∧(H~,C))

does not hold. By Corollary 19, we know that =_*J*_ is the weakest IVF soft equal relation. Thus, it follows that

(35) (F~,A)∧((G~,B)∨(H~,C))=L((F~,A)∧(G~,B))∨((F~,A)∧(H~,C)),(F~,A)∧((G~,B)∨(H~,C))≡((F~,A)∧(G~,B))∨((F~,A)∧(H~,C))

do not hold in general. In a similar fashion, one can verify that all the distributive inequalities in Theorems 40 and 41 described by ${\tilde{\subseteq }}_{L}$ cannot be replaced by distributive laws using any kinds of IVF soft equal relations including ≡, =_*L*_, and =_*J*_.

### 4.4. Absorptive Inequality of IVF Soft Sets

As mentioned above, the set (*I*(*U*), ∩, ∪) of all interval-valued fuzzy sets on *U* forms a distributive lattice. Thus, it is well known that the following types of absorptive laws are valid in the theory of interval-valued fuzzy sets.

**Proposition 44 .**

Let *μ*, *ν* ∈ *I*(*U*). Then we have

1. *μ*∩(*μ* ∪ *ν*) = *μ*;
2. *μ* ∪ (*μ*∩*ν*) = *μ*.

Considering IVF soft sets, we have the following result which shows that the absorptive law similar to the first assertion in Proposition 44 holds in a much weaker form characterized in terms of IVF soft *J*-equal relations.

**Theorem 45 .**

Let $\left(\tilde{F},A\right)$ and $\left(\tilde{G},B\right)$ be IVF soft sets over *U*. Then we have

(36) $$\begin{array}{c} \left(\tilde{F},A\right)\wedge \left(\left(\tilde{F},A\right)\vee \left(\tilde{G},B\right)\right){=}_{J}\left(\tilde{F},A\right). \end{array}$$

**Proof**

First, we denote $\left(\tilde{F},A)\vee (\tilde{G},B\right)$ by $\left(\tilde{T},A\times B\right)$, where $\tilde{T}(a,b)=\tilde{F}(a)\cup \tilde{G}(b)$ for all (*a*, *b*) ∈ *A* × *B*. Let $\left(\tilde{F},A)\wedge ((\tilde{F},A)\vee (\tilde{G},B))=(\tilde{L},A\times (A\times B)\right)$, where

(37) $$\begin{array}{c} \tilde{L}\left(a_{1},\left(a_{2},b\right)\right)=\tilde{F}\left(a_{1}\right)\cap \tilde{T}\left(a_{2},b\right)=\tilde{F}\left(a_{1}\right)\cap \left(\tilde{F}\left(a_{2}\right)\cup \tilde{G}\left(b\right)\right) \end{array}$$

for all (*a*_1_, (*a*_2_, *b*)) ∈ *A* × (*A* × *B*).

For any *a* ∈ *A*, there exists a parameter (*a*, (*a*, *b*)) ∈ *A* × (*A* × *B*) such that

(38) $$\begin{array}{c} \tilde{F}\left(a\right)=\tilde{F}\left(a\right)\cap \left(\tilde{F}\left(a\right)\cup \tilde{G}\left(b\right)\right)=\tilde{L}\left(a,\left(a,b\right)\right). \end{array}$$

This implies $\left(\tilde{F},A\right)\;{\tilde{\subseteq }}_{L}\;(\tilde{L},A\times (A\times B))$. Thus, it follows that

(39) $$\begin{array}{c} \left(\tilde{F},A\right){\tilde{\subseteq }}_{J}\left(\tilde{F},A\right)\wedge \left(\left(\tilde{F},A\right)\vee \left(\tilde{G},B\right)\right). \end{array}$$

Note also that the reverse IVF soft *J*-inclusion

(40) $$\begin{array}{c} \left(\tilde{F},A\right)\wedge \left(\left(\tilde{F},A\right)\vee \left(\tilde{G},B\right)\right){\tilde{\subseteq }}_{J}\left(\tilde{F},A\right) \end{array}$$

holds according to Proposition 25.

The above result is called the* weak absorptive law* of IVF soft sets. From its proof above, we can also obtain the following absorptive inequalities of IVF soft sets described by IVF soft *L*-inclusions.

**Proposition 46 .**

Let $\left(\tilde{F},A\right)$ and $\left(\tilde{G},B\right)$ be IVF soft sets over *U*. Then we have

(41) $$\begin{array}{c} \left(\tilde{F},A\right){\tilde{\subseteq }}_{L}\left(\tilde{F},A\right)\wedge \left(\left(\tilde{F},A\right)\vee \left(\tilde{G},B\right)\right). \end{array}$$

**Proposition 47 .**

Let $\left(\tilde{F},A\right)$ and $\left(\tilde{G},B\right)$ be IVF soft sets over *U*. Then we have

(42) $$\begin{array}{c} \left(\tilde{F},A\right){\tilde{\subseteq }}_{L}\left(\tilde{F},A\right)\vee \left(\left(\tilde{F},A\right)\wedge \left(\tilde{G},B\right)\right). \end{array}$$

It can easily be verified that the reverse IVF soft *L*-inclusions do not hold in general. Thus, we cannot replace =_*J*_ by =_*L*_ in Theorem 45.

More interesting, it is worth noting that the absorptive law of IVF soft sets similar to the second assertion in Proposition 44 might not hold even in the weakest sense of =_*J*_. To illustrate this, we consider an example as follows.

**Example 48 .**

Let $A=(\tilde{F},A)$ and $B=(\tilde{G},B)$ be the IVF soft sets given by Tables 1 and 5 (see Example 43). Let us write $\left(\tilde{M},A\times B\right)$ for $\left(\tilde{F},A)\wedge (\tilde{G},B\right)$, where $\tilde{M}(a,b)=\tilde{F}(a)\cap \tilde{G}(b)$ for all (*a*, *b*) ∈ *A* × *B*. It is clear that *A* × *B* = {(*e*_1_, *e*_3_), (*e*_2_, *e*_3_)} and by calculation, we get

(43) M~(e1,e3)=F~(e1)∩G~(e3)={(u1,[0.3,0.5]),(u2,[0.2,0.6]),(u3,[0.1,0.2]), (u4,[0.1,0.2]),(u5,[0.3,0.5]), (u6,[0.2,0.3])},

(44) M~(e2,e3)=F~(e2)∩G~(e3)={(u1,[0.2,0.5]),(u2,[0.1,0.4]),(u3,[0.3,0.5]), (u4,[0.1,0.2]),(u5,[0.4,0.5]), (u6,[0.2,0.3])}.

Then let us write $\left(\tilde{F},A)\vee (\tilde{M},A\times B\right)$ as $L=(\tilde{L},A\times (A\times B))$, where

(45) L~(a1,(a2,b))=F~(a1)∪M~(a2,b)=F~(a1)∪(F~(a2)∩G~(b))

for all (*a*_1_, (*a*_2_, *b*)) ∈ *A* × (*A* × *B*). It is easy to see that

(46) A×(A×B)={(e1,(e1,e3)),(e1,(e2,e3)), (e2,(e1,e3)),(e2,(e2,e3))}.

Proceeding with detailed calculations, one can obtain the IVF soft set *L* with its tabular representation given in Table 7.

Taking the parameter (*e*_1_, (*e*_2_, *e*_3_)) ∈ *A* × (*A* × *B*)), it can be verified that $\tilde{L}(e_{1},(e_{2},e_{3})\neq \tilde{F}(e_{1})$ and $\tilde{L}(e_{1},(e_{2},e_{3})\neq \tilde{F}(e_{2})$. Hence, by the definition of IVF soft *J*-inclusions, $\left(\tilde{L},A\times (A\times B))\;{\tilde{\subseteq }}_{J}\;(\tilde{F},A\right)$ does not hold and so we deduce that

(47) $$\begin{array}{c} \left(\tilde{F},A\right)\vee \left(\left(\tilde{F},A\right)\wedge \left(\tilde{G},B\right)\right)\neq_{J}\left(\tilde{F},A\right). \end{array}$$

On the other hand, one can verify the following fact

(48) $$\begin{array}{c} \left(\tilde{F},A\right)\wedge \left(\left(\tilde{F},A\right)\vee \left(\tilde{G},B\right)\right){=}_{J}\left(\tilde{F},A\right). \end{array}$$

In contrast to the absorption laws of interval-valued fuzzy sets given in Proposition 44, the above example and Theorem 45 further confirm that the soft product operations ∧ and ∨ of IVF soft sets do not always have similar algebraic properties as mentioned in Remark 39.

## 5. Conclusions

This study has been devoted to exploring some nonclassical algebraic properties of IVF soft sets under the soft product operations. Some algebraic inequalities of IVF soft sets characterized by IVF soft inclusions have been obtained and the relations among four different types of interval-valued fuzzy soft subsets have been ascertained. It has been shown that the commutative, associative, idempotent, and absorptive laws of IVF soft sets do not hold in the conventional sense described by IVF soft identical relations but hold in some weaker forms characterized in terms of the IVF soft equal relations =_*L*_ or =_*J*_. We have found that the soft product operations ∧ and ∨ of IVF soft sets do not always have similar algebraic properties and only certain types of distributive inequalities instead of distributive laws hold for IVF soft sets. Some of the results obtained here extend the corresponding results for Molodtsov's soft sets in [29, 30]. As future work, we will further investigate free IVF soft algebras. It will be also interesting to consider potential applications of different types of interval-valued fuzzy soft subsets in decision making under uncertainty.

## Figures and Tables

**Table 1:** Tabular representation of the IVF soft set *A*.

*U*	*e*_1_	*e*_2_
*u*_1_	[0.4,0.7]	[0.2,0.6]
*u*_2_	[0.2,0.6]	[0.1,0.4]
*u*_3_	[0.1,0.2]	[0.5,0.7]
*u*_4_	[0.8,0.9]	[0.6,0.9]
*u*_5_	[0.3,0.5]	[0.6,0.7]
*u*_6_	[0.4,0.6]	[0.4,0.5]

**Table 2:** Tabular representation of the IVF soft set *B*_1_.

*U*	*e*_1_	*e*_2_	*e*_3_
*u*_1_	[0.5,0.7]	[0.3,0.7]	[0.5,0.6]
*u*_2_	[0.3,0.7]	[0.4,0.6]	[0.8,0.9]
*u*_3_	[0.5,0.6]	[0.6,0.8]	[0.2,0.3]
*u*_4_	[0.8,0.9]	[0.7,0.9]	[0.2,0.4]
*u*_5_	[0.3,0.6]	[0.6,0.7]	[0.6,0.7]
*u*_6_	[0.5,0.6]	[0.4,0.6]	[0.4,0.7]

**Table 3:** Tabular representation of the IVF soft set *B*_2_.

*U*	*e*_2_	*e*_3_	*e*_4_
*u*_1_	[0.2,0.6]	[0.5,0.7]	[0.5,0.6]
*u*_2_	[0.1,0.4]	[0.3,0.7]	[0.8,0.9]
*u*_3_	[0.5,0.7]	[0.5,0.6]	[0.2,0.3]
*u*_4_	[0.6,0.9]	[0.8,0.9]	[0.2,0.4]
*u*_5_	[0.6,0.7]	[0.6,0.7]	[0.6,0.7]
*u*_6_	[0.4,0.5]	[0.4,0.6]	[0.4,0.7]

**Table 4:** Tabular representation of the IVF soft set *B*_3_.

*U*	*e*_2_	*e*_3_	*e*_4_
*u*_1_	[0.2,0.6]	[0.4,0.7]	[0.4,0.7]
*u*_2_	[0.1,0.4]	[0.2,0.6]	[0.2,0.6]
*u*_3_	[0.5,0.7]	[0.1,0.2]	[0.1,0.2]
*u*_4_	[0.6,0.9]	[0.8,0.9]	[0.8,0.9]
*u*_5_	[0.6,0.7]	[0.3,0.5]	[0.3,0.5]
*u*_6_	[0.4,0.5]	[0.4,0.6]	[0.4,0.6]

**Table 5:** Tabular representation of the IVF soft set *B*.

*U*	*e*_3_
*u*_1_	[0.3,0.5]
*u*_2_	[0.4,0.8]
*u*_3_	[0.3,0.5]
*u*_4_	[0.1,0.2]
*u*_5_	[0.4,0.5]
*u*_6_	[0.2,0.3]

**Table 6:** Tabular representation of the IVF soft set *C*.

*U*	*e*_4_
*u*_1_	[0.3,0.5]
*u*_2_	[0.2,0.5]
*u*_3_	[0.3,0.5]
*u*_4_	[0.7,0.9]
*u*_5_	[0.2,0.6]
*u*_6_	[0.5,0.6]

**Table 7:** Tabular representation of the IVF soft set *L*.

*U*	(*e*_1_, (*e*_1_, *e*_3_))	(*e*_1_, (*e*_2_, *e*_3_))	(*e*_2_, (*e*_1_, *e*_3_))	(*e*_2_, (*e*_2_, *e*_3_))
*u*_1_	[0.4,0.7]	[0.4,0.7]	[0.3,0.6]	[0.2,0.6]
*u*_2_	[0.2,0.6]	[0.2,0.6]	[0.2,0.6]	[0.1,0.4]
*u*_3_	[0.1,0.2]	[0.3,0.5]	[0.5,0.7]	[0.5,0.7]
*u*_4_	[0.8,0.9]	[0.8,0.9]	[0.6,0.9]	[0.6,0.9]
*u*_5_	[0.3,0.5]	[0.4,0.5]	[0.6,0.7]	[0.6,0.7]
*u*_6_	[0.4,0.6]	[0.4,0.6]	[0.4,0.5]	[0.4,0.5]
